# Chondroblastoma of the knee in a teenager

**DOI:** 10.1016/j.radcr.2021.08.065

**Published:** 2021-10-02

**Authors:** Maitham Alkadumi, Neil Duggal, Sukhman Kaur, Joseph Dobtsis

**Affiliations:** Department of Radiology, NYC Health + Hospital / Harlem, New York, USA

**Keywords:** Chondroblastoma, Chondroblastoma of the knee in a teenager, Pediatric musculoskeletal tumor

## Abstract

Chondroblastoma is an uncommon osseous neoplasm that accounts for less than 1% of all bone tumors. Characteristically it arises in the epiphysis or apophysis of long bones in young patients and may extend into the metaphysis. A sixteen-year-old male presents with a 1-year history of left knee pain associated with joint stiffness and interfering with performing daily activities. Radiographic and contrast enhanced magnetic resonance imaging favored the diagnosis of chondroblastoma. This was then confirmed histologically. The clinical signs and symptoms of Chondroblastoma are usually nonspecific, pain is most often moderate and can be revealed as a result of a trauma. The definitive diagnosis is mainly pathological due to the lack of specificity of radiological findings consistent with the presented case.

## Introduction

Chondroblastoma presented a puzzling classification conundrum for early physicians. First remarked on by Ewing as a “calcifying” giant cell tumor associated with cartilage resorption generally at the humeral head and then by Codman in his review of nine cases from the Bone Sarcoma Registry as “epiphyseal chondromatous” giant cell tumors [15,16]. Both classifications recognize these lesions as a unique entity but under the broader heading of giant cell tumor. It was Jaffe and Lichenstein who first recognized chondroblastoma as an entity from giant cell tumors due to its separate histologic features with focal areas of calcification, areas of necrosis, and collagenization of these necrotic areas with hyaline chondroid tissue. They note that while it is true chondroblastoma displays multinucleated giant cells characteristic of giant cell tumors, but they are only seen “here and there” and not the dominant histologic feature of chondroblastoma [1].

## Case report

A sixteen-year-old male presents with a 1-year history of left knee pain associated with joint stiffness and interfering with performing daily activities. Radiographic and contrast enhanced magnetic resonance imaging favored the diagnosis of chondroblastoma. This was then confirmed histologically.

**Fig. 1:**
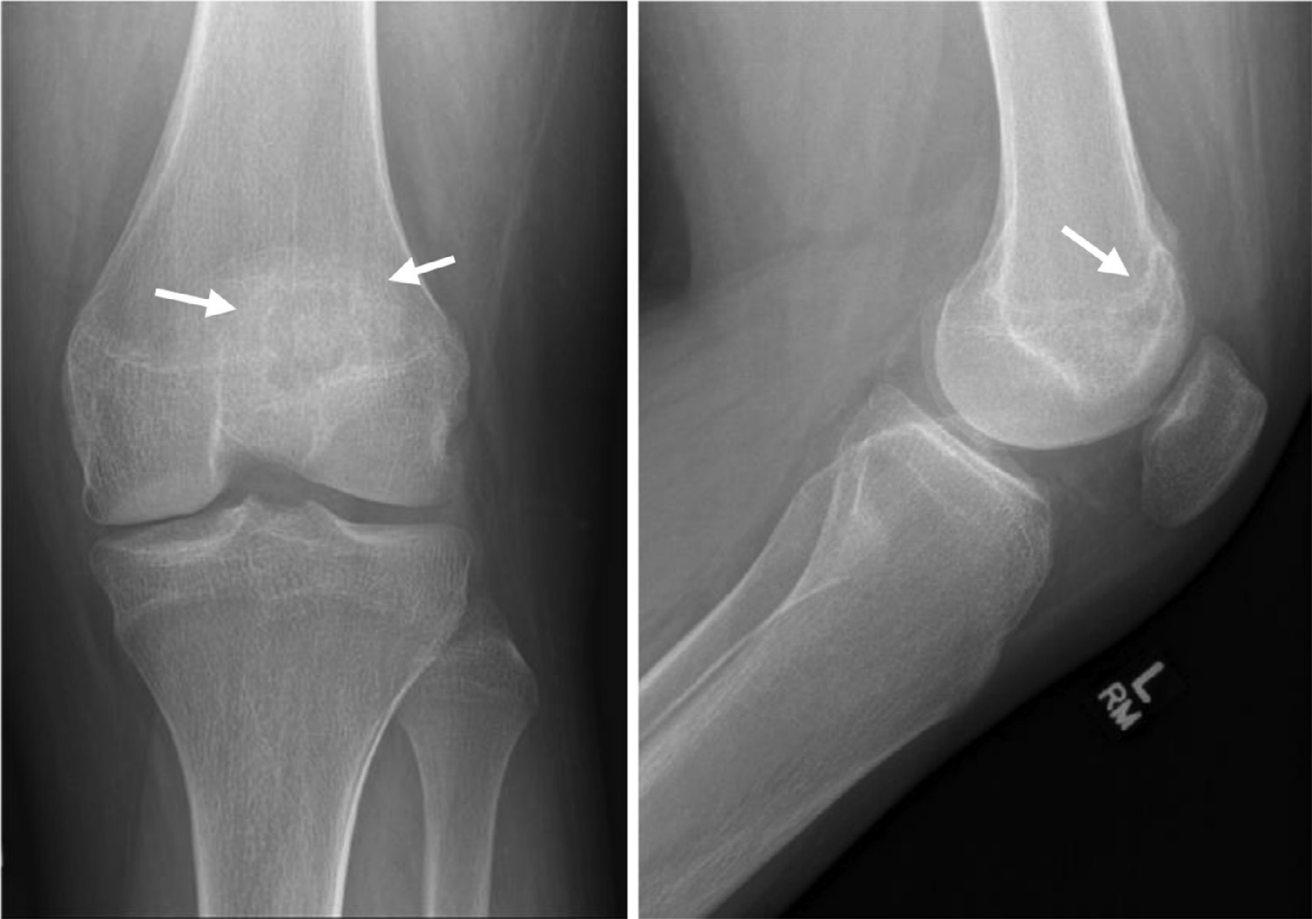
AP and lateral radiographs of the knee showing a very subtle radiolucent lesion in the distal femoral epiphysis involving the physis and metaphysis. The lesion has faint sclerotic margin (white arrows)

**Fig. 2:**
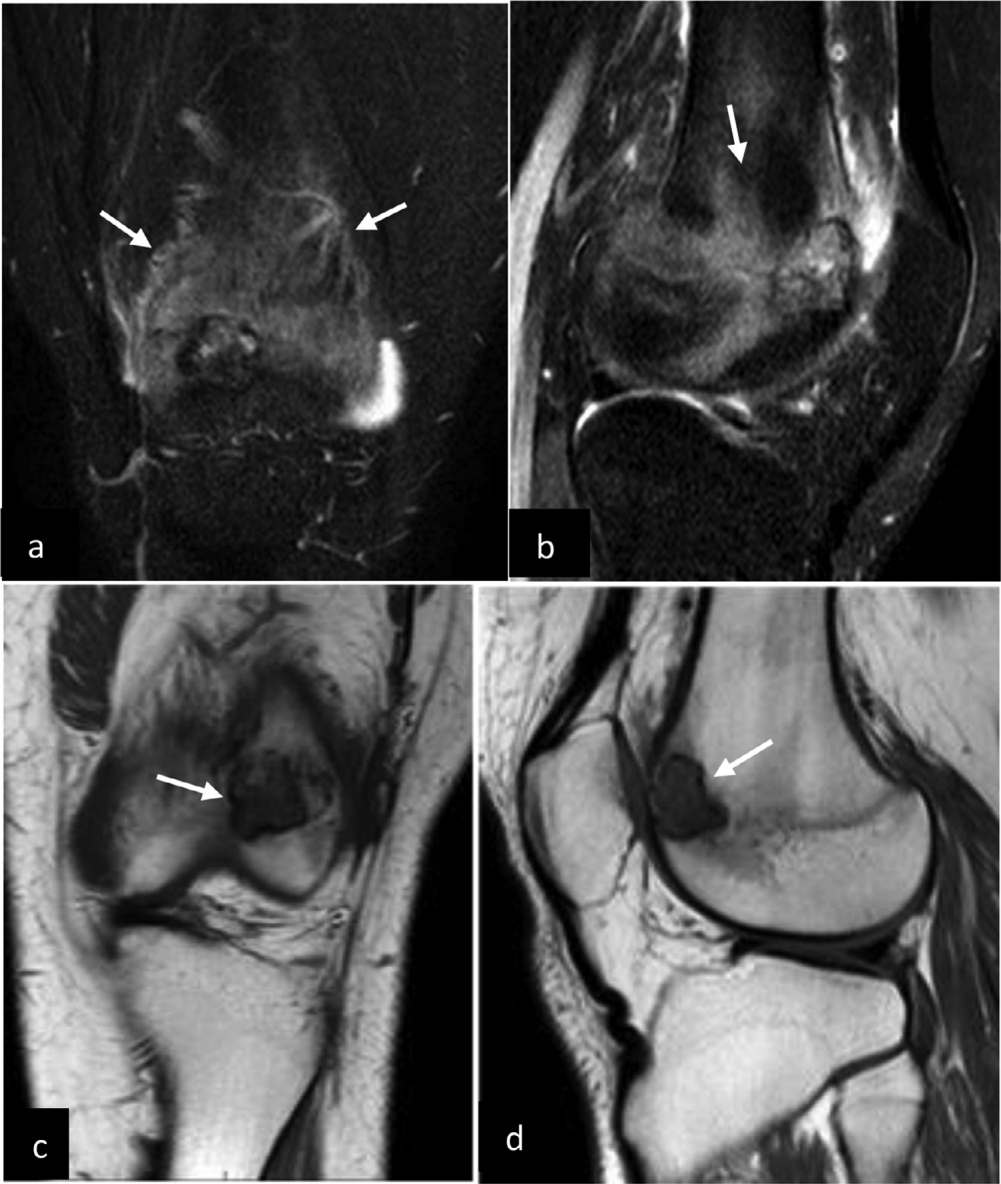
STIR images (A) and (B) demonstrating extensive edema (white arrows) in the surrounding bone as well as mild periosteal reaction. T1WI coronal (C) and sagittal (D) images demonstrate the lesion (white arrows) within the femoral epiphysis extending into the physis and metaphysis. The lesion demonstrates low T1 signal and thin hypointense margin. There is slight erosion of the overlying cortex

**Fig. 3:**
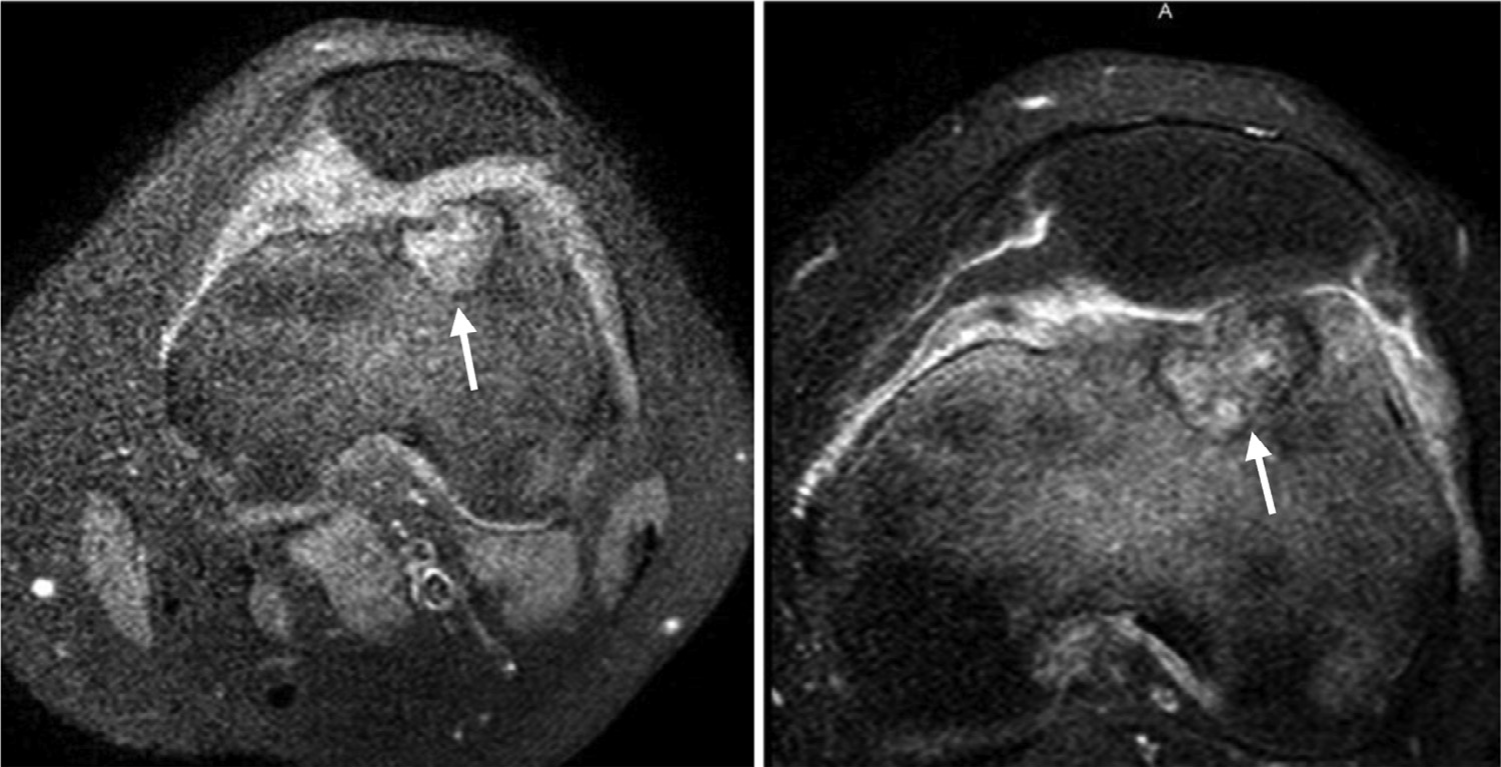
Axial Fat suppressed T1W pre- and post-contrast images demonstrate T1 hyper intense signal with indeterminate enhancement pattern (white arrows). There is reactive small joint effusion

The patient is a basketball player, however since onset of the knee pain, he has not played and avoids physical activity. Physical examination of the left knee reveals diffuse tenderness and moderate sized joint effusion.  There is no fever or any other sign of systemic illness and laboratory findings were normal. Plain radiographs exhibit a lucent rounded lesion with sharp sclerotic margin within the distal anterior femoral metaphysis and epiphysis. (Fig. 1). MRI of the knee with IV contrast reveals a lesion located at the distal left femoral epiphysis extending through physes to the distal femoral metaphysis (Fig. 2).The lesion exhibits T2 hyper-intense signal and T1 low signal intensity with extensive surrounding bone marrow edema and slight adjacent periosteal reaction. The enhancement pattern is indeterminate as the lesion is hyperintense on both pre and post contrast T1 fat-suppressed sequences (Fig. 3). The location and morphology of this lesion as well as the age of the patient is suggestive of chondroblastoma. The main differential would be bone abscess, which is deemed less likely in the given clinical context. Curettage biopsy is performed, and histopathological investigation confirms the diagnosis of chondroblastoma. Patient is treated with intralesional curettage and bone graft.

## Discussion

Chondroblastoma is a rare type of benign cartilage tumor that accounts for approximately 1% of all bone tumors. It most frequently affects the skeletally immature so it is mostly encountered in children and young adults between ages 19 to 23 with a M>F ratio of 2:1 [2]. The predilection for the skeletally immature helps to differentiate chondroblastoma from giant cell tumors which usually occur in older patients with closed physes. Chondroblastoma most frequently affects the epiphysis or apophysis of long bones.[3], [4], [5], [6]. However, with growth the lesion may extend into the metaphysis. Additionally, periosteal reaction and edema is associated with these lesions helping to differentiate them from clear cell chondrosarcoma [18] Periosteal reaction of chondroblastoma is characterized by distinctive thick solid or layered periosteal response along the metaphyseal shaft distal to lesion [17,18]. The most common anatomical sites involved are the proximal humerus then the distal femur, proximal femur, proximal tibia, talus and innominate (hip) bone, in descending order. [7]

Generally, the presentation is nonspecific with common presenting symptoms including mild localized pain, swelling, and loss of range of motion of the affected joint. This is thought to be the result of chondroblastoma's production of prostaglandins [8,19]. Chondroblastoma is associated with aneurysmal bone cysts, especially when presenting in the patella. While generally considered benign, malignant transformation has been rarely reported, metastasizing to the lung and may be the result of incorrect original diagnosis or secondary to previous radiation in individual case reports [20,21].

Typically, on radiograph chondroblastoma presents with an eccentrically or centrally located osteolytic lesion with either smooth or lobulated margins and thin sclerotic rim involving the epiphysis or other secondary ossification centers. [9] In 25-50% of the cases metaphyseal involvement is also seen with growth. [10] Cortical expansion with erosion and periosteal reaction and edema may be present. Typically, this is seen in more longstanding lesions with up to one half developing thick smooth periosteal reaction as described above. Presence of adjacent edema helps distinguish it from the most common differential of giant cell tumor but is not helpful in differentiating from another common differential of clear cell chondrosarcoma which is often associated with adjacent edema. Up to one half contain a chondroid matrix. On cross sectional imaging, CT findings mimic radiographic appearance with better visualization of chondroid matrix. MRI findings show characteristic low T1 with in homogeneously high intensity on fluid sensitive sequences. Inhomogeneity is thought to be secondary to the combination of chondroid matrix, calcification, and fluid. High signal on fluid sensitive sequences adjacent to cortex, in marrow, and in soft tissue usually represents edema and correlates with degree of periosteal reaction. [11,23].

Chondroblastoma is nonprogressive if left in situ. Treatment depends on the anatomic location of the lesion and the extent of bone and/or joint involvement. It is usually treated by curettage and bone graft. [12,^13^,14]. This procedure is curative in 90% of the cases. Other methods such as curettage alone, endoscopic curettage, endoscopic curettage with cementation, curettage with fat implantation, resection with allograft replacement, marginal resection radiofrequency ablation and osteochondral autograft transfer have been used with some success. [14] Recurrence has been noted in up to 10% of chondroblastomas [22].

## Conclusion

Chondroblastoma is a rare type of benign cartilage tumor that accounts for approximately 1% of all bone tumors. It most frequently affects children and young adults. The clinical signs and symptoms are usually nonspecific, pain is most often moderate and can be revealed as a result of a trauma. The definitive diagnosis is mainly pathological due to the lack of specificity of radiological findings consistent with the presented case. The treatment is almost always surgical curettage and bone grafting. The functional prognosis of chondroblastoma depends on its location and its degree of aggressiveness as defined by joint destruction and nearby bony extension.
